# Photodynamic Therapy as a Novel Therapeutic Modality Applying Quinizarin-Loaded Nanocapsules and 3D Bioprinting Skin Permeation for Inflammation Treatment

**DOI:** 10.3390/ph17091169

**Published:** 2024-09-04

**Authors:** Stéphanie R. do Amaral, Camila F. Amantino, Aleksandar Atanasov, Stefanie Oliveira Sousa, Richard Moakes, Sonia Maria Oliani, Liam M. Grover, Fernando L. Primo

**Affiliations:** 1 Department of Bioprocess and Biotechnology Engineering, School of Pharmaceutical Sciences, São Paulo State University (UNESP), Araraquara 14800-903, SP, Brazil; stephanie.amaral@unesp.br (S.R.d.A.); camila.amantino@unesp.br (C.F.A.); 2 São Paulo Federal Institute of Education, Science and Technology (IFSP), Matão 15991-502, SP, Brazil; 3 School of Chemical Engineering, University of Birmingham, Birmingham B15 2TT, UK; axa1712@student.bham.ac.uk (A.A.); r.j.a.moakes@bham.ac.uk (R.M.); l.m.grover@bham.ac.uk (L.M.G.); 4 Department of Biology, Institute of Biosciences, Humanities and Exact Sciences (IBILCE), São Paulo State University (UNESP), São José do Rio Preto 15054-000, SP, Brazil; stefanie.sousa@unesp.br (S.O.S.); sonia.oliani@unesp.br (S.M.O.)

**Keywords:** skin inflammation, photodynamic therapy, nanomedicine

## Abstract

Skin inflammation associated with chronic diseases involves a direct role of keratinocytes in its immunopathogenesis, triggering a cascade of immune responses. Despite this, highly targeted treatments remain elusive, highlighting the need for more specific therapeutic strategies. In this study, nanocapsules containing quinizarin (QZ/NC) were developed and evaluated in an in vitro model of keratinocyte-mediated inflammation, incorporating the action of photodynamic therapy (PDT) and analyzing permeation in a 3D skin model. Comprehensive physicochemical, stability, cytotoxicity, and permeation analyses of the nanomaterials were conducted. The nanocapsules demonstrated desirable physicochemical properties, remained stable throughout the analysis period, and exhibited no spectroscopic alterations. Cytotoxicity tests revealed no toxicity at the lowest concentrations of QZ/NC. Permeation and cellular uptake studies confirmed QZ/NC permeation in 3D skin models, along with intracellular incorporation and internalization of the drug, thereby enhancing its efficacy in drug delivery. The developed model for inducing the inflammatory process in vitro yielded promising results, particularly when the synthesized nanomaterial was combined with PDT, showing a reduction in cytokine levels. These findings suggest a potential new therapeutic approach for treating inflammatory skin diseases.

## 1. Introduction

The skin is the largest organ in the human body. It performs vital protective functions and has three layers: the epidermis, dermis, and the subcutaneous tissue. The epidermis is the outermost layer and is mainly made up of keratinocytes (95%), as well as cells such as melanocytes, Langerhans cells, and Merkel cells. Keratinocytes are fundamental cells in the skin’s inflammatory process and related immune responses, interacting with immune system cells by releasing cytokines and chemokines [1,2,3]. 

Epithelial cells are influenced by intrinsic genetic factors, as well as by the environment and the microbiome, which can have a direct influence on immune responses to potential allergens and pathogens. During an inflammatory response, keratinocytes release pro-inflammatory cytokines (TNF-α, IL-1α, IL-1β, IL-18, and IL-1), inducing the expression of new inflammatory cytokines (IL-6, IL-8, and TNF-α) and adhesion molecules on endothelial cells, leading to the targeting of effector T cells to the site of inflammation, and each type of injury/inflammation leads to the different activation and recruitment of the most convenient cell subset. Uncontrolled immune responses can lead to chronic inflammatory diseases such as psoriasis and atopic dermatitis [4,5]. 

Psoriasis, atopic dermatitis, and rosacea are the most common chronic skin diseases, characterized by hyperproliferation of keratinocytes, involving multifactorial inflammatory responses linked to highly complex autoimmune responses [6,7]. Psoriasis is characterized by excessive growth and the abnormal differentiation of keratinocytes, leading to skin lesions, linked to increased expression of immune cells and inflammatory cytokines. Currently, 2–4% of the world’s population is affected, giving antipsoriatic drugs a market worth close to 18.8 billion USD in 2021 [8,9,10]. The inflammatory mechanism of psoriasis involves the presence of keratinocytes, leukocytes, T cells, macrophages, neutrophils, mast cells, and dendritic cells, followed by the release of inflammatory cytokines [11]. Keratinocytes are activated by pro-inflammatory cytokines such as TNF-α and IL-1, which are elevated in psoriatic lesions. Once activated, keratinocytes change their proliferation and differentiation, thickening the epidermis and leading to increased production of chemokines, leading to a positive feedback loop between keratinocytes and immune system cells [12].

Considering psoriasis treatment, the biggest obstacle is the physiology of the skin, which becomes stiffer and more impermeable to medicines, limiting their bioavailability at the lower epidermis [10]. The first line of treatment for psoriasis and other chronic skin diseases consists of topical therapy. If this treatment is no longer effective, it is preceded with phototherapy and systemic drug administration [9]. However, these methods are associated with numerous side effects, such as nausea, irritation of the skin, and hepatotoxicity, in addition to drug resistance. Combined, these factors define the need for more effective treatments, with greater drug targeting and bioavailability [13,14].

Photodynamic therapy (PDT) is a widely used therapeutic modality, with promising results in patients with various types of superficial skin diseases such as psoriasis [15]. Contrary to biological agents and other medications, PDT is a powerful and secure technique with no systemic side effects [16]. The mechanism of action of PDT is based on photochemistry-controlled reactions, which can induce changes in the cellular metabolism, including induction of cell death via apoptosis and/or necrosis based on the cell type, light intensity, and PS type [9,17,18]. One of the biggest challenges of PDT is the choice of the most suitable photosensitizer, which has essential characteristics such as biological affinity, biodistribution, and tissue penetration [15]. Quinizarin (QZ) (1,4 dihydroxyanthraquinone), an anthraquinone derivative compound, shows secondary biological activity characteristics such as anti-inflammatory, antioxidant, and antibacterial action [19,20,21], and high potential as photosensitizing compounds [22], showing high-density planar and quinoid-conjugated aromatic group electronics, which may contribute to the generation of reactive oxygen species (ROS), [23,24,25,26], with a high potential as photosensitizing compounds from a PDT perspective [22].

However, there are some limitations to the use of anthraquinones in PDT, as their low solubility in aqueous media can cause a lack of selectivity in the treatment. Pharmaceutical nanotechnology is a valuable resource, which can significantly influence the use of anthraquinone derivatives in PDT, increasing their permeation at the required site, as well as their controlled and sustained release [27]. Among the main colloidal nanocarriers with potential application, the polymeric and emulsified systems come into focus, especially polymeric nanocapsules (NCs). They are mostly composed of biodegradable polymers, are biocompatible, avoiding toxicity, and presenting high efficiency of encapsulation [28]. In addition, the polymeric wrapping present in nanocapsules preserves the drug from the degradative effects of factors such as light, oxygen, and the acidic stomach environment, and controls the release of the internalized active into the biological targets of interest [29]. 

The development of new therapies has been underpinned for years by two-dimensional culture (monolayer) or animal models, but limitations linked to reliability and reproducibility still exist. The concept of tissue engineering has evolved rapidly in this context, opening space for three-dimensional printing, an automated and highly efficient technology for creating porous and complex structures, capable of reliable systems for drug testing and disease modeling [30]. Technologies such as the suspended layer additive manufacture (SLAM) method aim to overcome the limitations of 3D bioprinting cell-laden constructs. The technology presents a solution for creating hydrogels with defined properties, making it a valuable tool for recapitulating in vivo interactions, mimicking the complexity of real tissues [31,32,33]. 

The present study proposes the development and characterization of a 2D model of an inflammatory process in human keratinocyte cells for the application of PDT using polymeric QZ-loaded nanocapsules, an anthraquinone derivative with secondary biological activities, evaluating the joint action of nanotechnology and PDT in a new therapeutic modality, combined with tissue engineering. 

## 2. Results

### 2.1. Physical–Chemical and Two-Dimensional Topography Analysis: Particle Size, PdI, Zeta Potential, and AFM

Stability studies are essential for the pharmaceutical and industrial application of nanomaterials [34]. The particle size, polydispersity index (PdI), and zeta potential (ζ), which provides surface charge, were determined using the dynamic light scattering (DLS) technique. The average of the particle size values obtained for QZ/NC and for Unloaded/NC were expressed as mean ± standard deviation and are shown in Table 1 after 61 days of synthesis. Both synthesized nanomaterials (QZ/NC and Unloaded/NC) showed a satisfactory profile, considering the particle size and zeta potential. The PdI values suggest moderated polydispersity; however, 97.9% of the particles remain in the predominant average size, indicating a monodisperse system with variations. 

**Figure 1:**
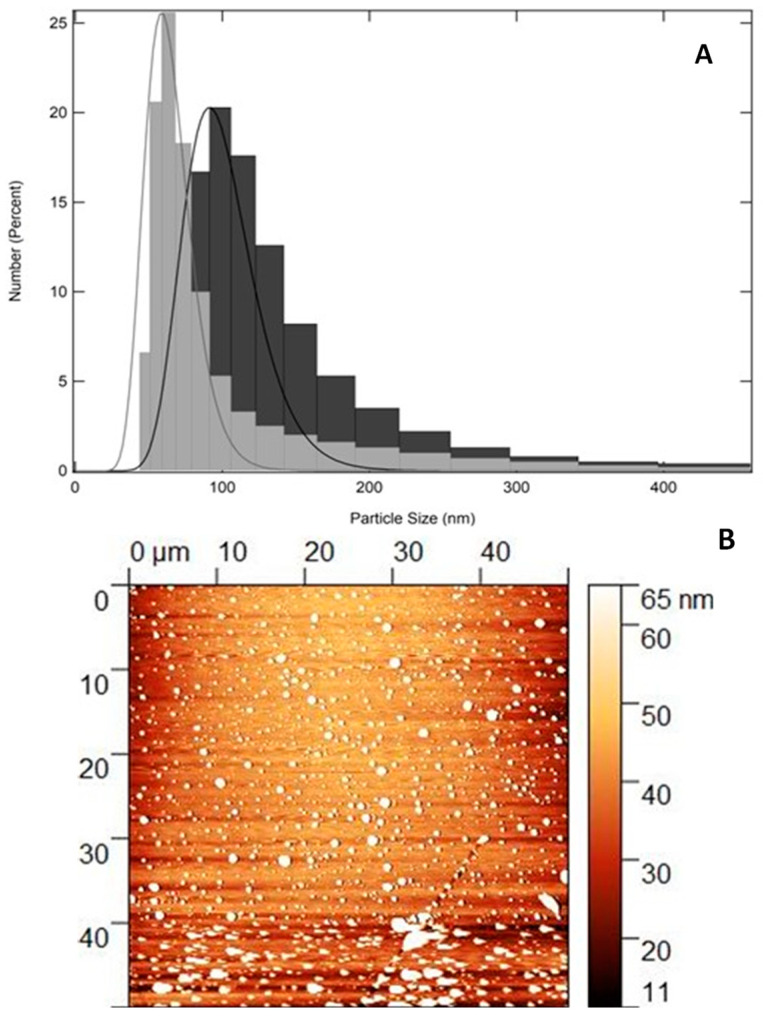
(**A**): Size distribution profile of the developed NC using dynamic light scattering technique. Unloaded/NC (gray) and QZ/NC (black) at 61 days after synthesis. (**B**): Two-dimensional photomicrograph of QZ/NC obtained using the AFM technique.

The AFM analysis of the QZ/NC, shown in Figure 1B, shows clear images of particles with a mostly spherical shape, with a size within the expected, around 65 nm. 

### 2.2. Three-Dimensional Fluorescence Emission Spectroscopy UV/Vis

The analysis of compounds is fundamental, facilitating their characterization and the identification of possible modifications. The fluorescence method, when compared to other spectral methods, has shown significant advantages, due to its simplicity, speed, and high sensitivity, with emphasis on 3D fluorescence spectroscopy, which measures emission spectra over a range of excitation wavelengths, allowing a more extensive identification of samples, becoming a powerful tool in the classification and identification of substances [35,36].

**Figure 2:**
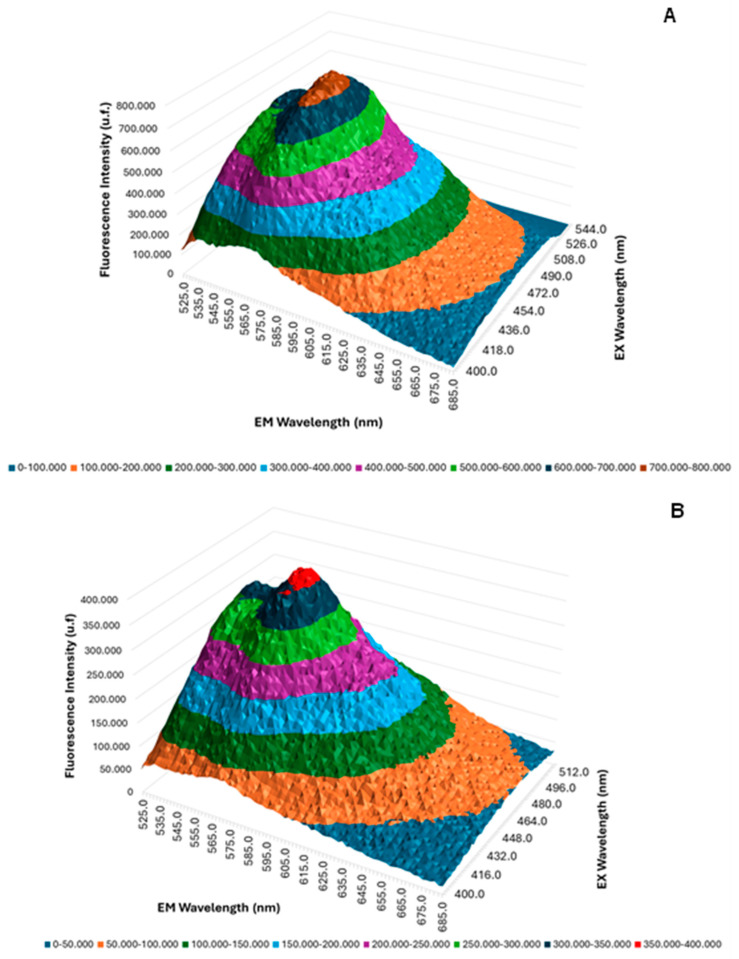
Three-dimensional fluorescence spectra of free QZ (**A**) and QZ/NC (**B**).

The 3D fluorescence emission spectra profiles of free QZ and QZ/NC were obtained by UV/Visible fluorescence emission spectroscopy studies, as shown in Figure 2, using acetonitrile as the organic solvent at the concentration of 8 µg/mL for free QZ and QZ/NC. The fluorescence intensity maximum profile was obtained at 569 nm from the excitation at 480 nm, with no significant difference inside the spectral profile between the free QZ and the nanocapsule loaded with QZ. 

### 2.3. Cytotoxicity Assay Using Resazurin Test—NIH/3T3 Cells

NIH/3T3 cells were used as preliminary assays to analyze the cytotoxicity of the nanocapsules and free QZ, assessing the material safety, as shown in Table 2. QZ/NC was analyzed at concentrations of 2.5–70 μg.mL^−1^, Unloaded/QZ 50% (*v*/*v*), and free QZ (50 and 70 μg.mL^−1^). These data are in accordance with the incubation times suggested in the international standard ISO 10993-5 [37] for the cytotoxicity testing of nanomaterials. 

The results developed with NIH/3T3 cells have shown the absence of cytotoxicity at the lower concentrations of QZ/NC, Unloaded/NC, and free QZ at both concentrations tested. At the higher concentrations of QZ/NC (50 µg.mL^−1^ and 70 µg.mL^−1^), there was a decrease in cell viability of approximately 30% and 90%, respectively, evidenced by the statistical significance when compared with the control.

### 2.4. Cell Internalization and Permeation Study

**Figure 3:**
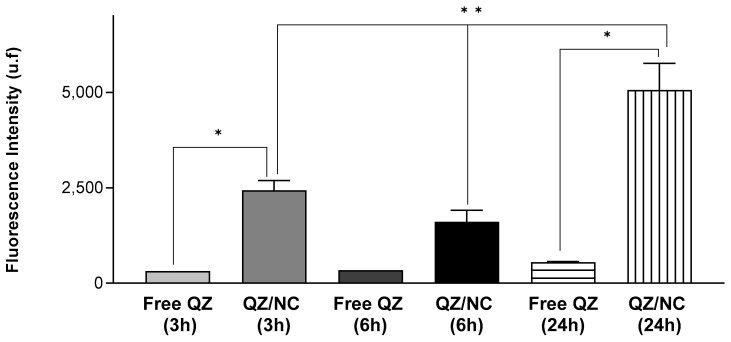
Cellular uptake study in HaCaT cells at a concentration of 15 μg.mL^−1^ evaluating the cellular internalization of free QZ and QZ-loaded nanocapsules (QZ/NC) at 3, 6, and 24 h. Statistical significance was determined using the one-way ANOVA test, with 95% significance level, followed by Tukey post-test for multiple comparisons, where * statistical difference obtained between free QZ and QZ/NC, ** statistical difference obtained between QZ/NC in the three tested times (*p* < 0.05).

In order to determine the internalization of QZ/NC and free QZ in HaCaT cells, three different incubation times (3, 6, and 24 h) were analyzed, at the concentration of 15 μg.mL^−1^. Higher internalization was observed for QZ/NC when compared to free QZ (Figure 3), demonstrating the desired effect of the increased cytoplasmic internalization of the nanocarrier active. 

Another data obtained in the assay were the best incubation time relative to the best internalization rates of the active. According to the following results, the time of 24 h presented a fluorescence intensity of QZ much higher than the previous times, indicating an internalization 9× higher than the others. These data are in accordance with the incubation times suggested in the international standard ISO 10993-5 for the cytotoxicity testing of nanomaterials.

**Figure 4:**
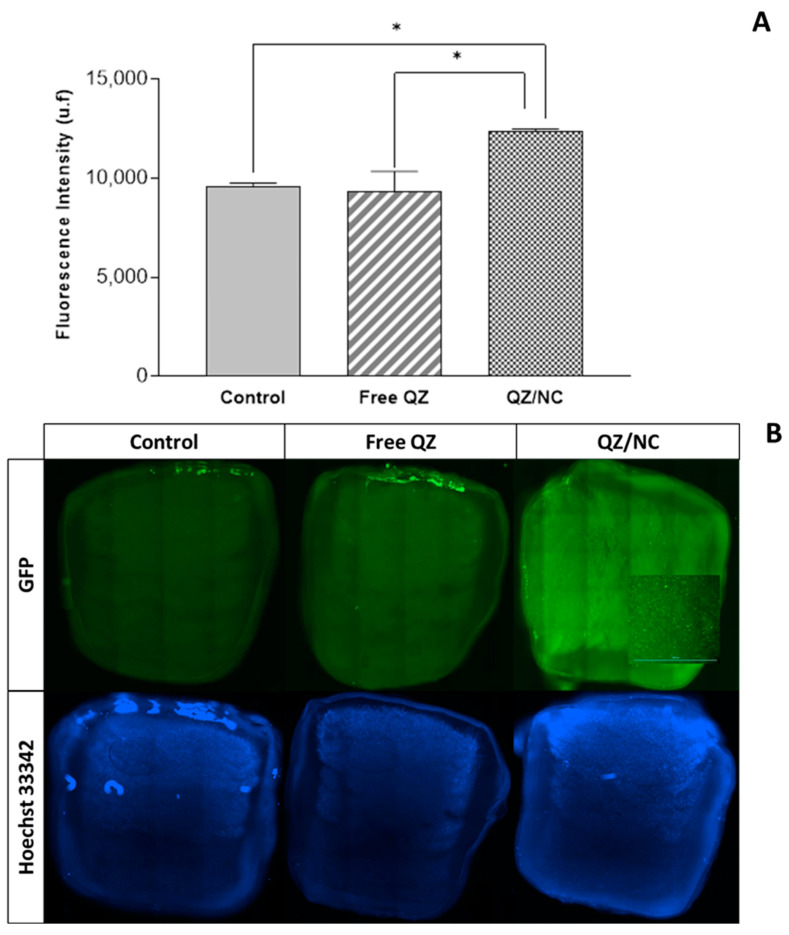
Analysis of permeation in the three-dimensional skin model by evaluating fluorescence intensity (**A**) and cross-sectional images and (**B**) using a SPARK microplate reader (TECAN, SWI) —scale bar indicates 3000 µm. The control, containing only DMEM, free QZ, and QZ/NC were analyzed. Statistical significance was determined using one-way ANOVA, at 95% significance level, followed by Tukey post-test for multiple comparisons (* *p* < 0.05).

The permeation of QZ/NC and free QZ was evaluated using a 3D bioprinted skin equivalent as a model, developed by the suspended layer additive manufacturing (SLAM) method, where computer-aided design (CAD) was used to create the design of the three layers. The composition of the bioink consisted of a mixture of pectin, which is similar to the polysaccharides found in native ECM, and collagen, mimicking the composition of skin [33]. Analyses were conducted to assess permeation after 24 h, as shown in Figure 4, in cross-sections (from top to bottom), using the previously determined excitation and fluorescence emission parameters of QZ (λex = 480 nm; λem = 569 nm). The concentration used was 15 µg.mL^−1^, defined in the cytotoxicity experiment as the drug’s safety threshold. The fluorescence signal of QZ/NC was observed in all the layers of the three-dimensional model, demonstrating the permeation.

### 2.5. Cytotoxicity Assay Using Resazurin Test—HaCaT Cells

The modulation of the human epidermis using monolayer keratinocytes has been studied extensively, making it possible to understand the functional characteristics of interactions within the cell [38]. An additional cell viability test was carried out on human keratinocyte cells (HaCaT), assessing the toxicity of QZ/NC at different concentrations (2.5–50 µg.mL^−1^), since the keratinocyte lineage was used to modulate the inflammatory process in vitro. The incubation time used was 24 h, as determined in Section 4.4.

**Figure 5:**
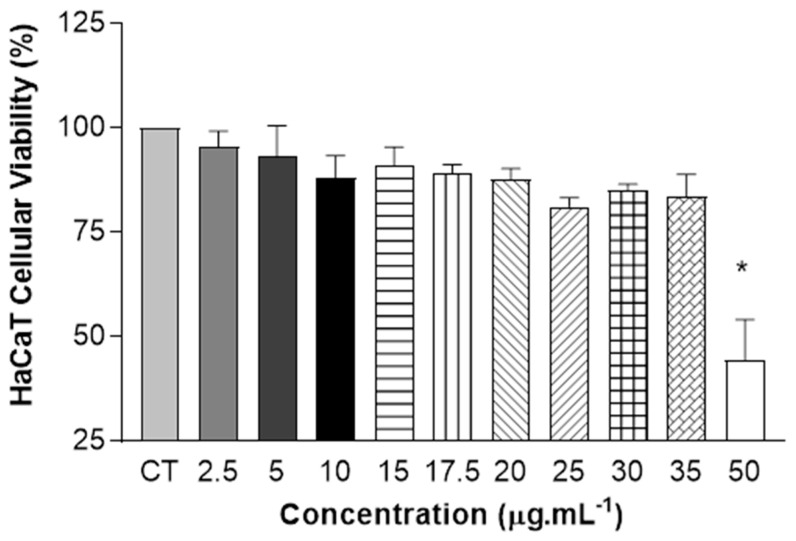
Safety tests from in vitro cytotoxicity assay of QZ/NC on human keratinocyte HaCaT (HaCaT—cell line service) at concentrations of 2.5; 5; 10; 15; 17.5; 20; 25; 30; 35; and 50 μg.mL^−1^ (QZ/NC). Statistical significance was determined using one-way ANOVA, at 95% significance level, followed by Tukey post-test for multiple comparisons (* *p* < 0.05).

As shown in Figure 5, the results of the one-way ANOVA statistical analysis show the absence of cytotoxicity for concentrations between 2.5 µg.mL^−1^ and 35 µg.mL^−1^, with cell viability above 70%. The reduction in viability was only observed at the highest concentration of QZ/NC tested.

### 2.6. Induction of Inflammatory Process in Human Keratinocytes with Application of Photodynamic Therapy and Immunoenzymatic Assay

After the induction of the inflammatory process, further analyses were performed using the enzyme-linked immunosorbent assay—ELISA—described in 4.10. Cytokines are small proteins secreted in cascade by cells in response to an inflammatory process, influencing interactions and communication between cells. The following pro-inflammatory cytokines were analyzed in this study: interleukin IL-1β (pro-inflammatory cytokine essential in the inflammatory response and defense of cells, and exacerbates damage in chronic diseases and acute tissue injury) [39]; interleukin IL-8 (chemokine responsible for the migratory stimulation of neutrophils and other immune cells to the site of inflammation [40], in addition to stimulating the cell proliferation of keratinocytes) [41]; and monocyte chemotactic protein-1 MCP-1 (responsible for regulating the release of monocytes/macrophages at the site of inflammation) [42]. 

**Figure 6:**
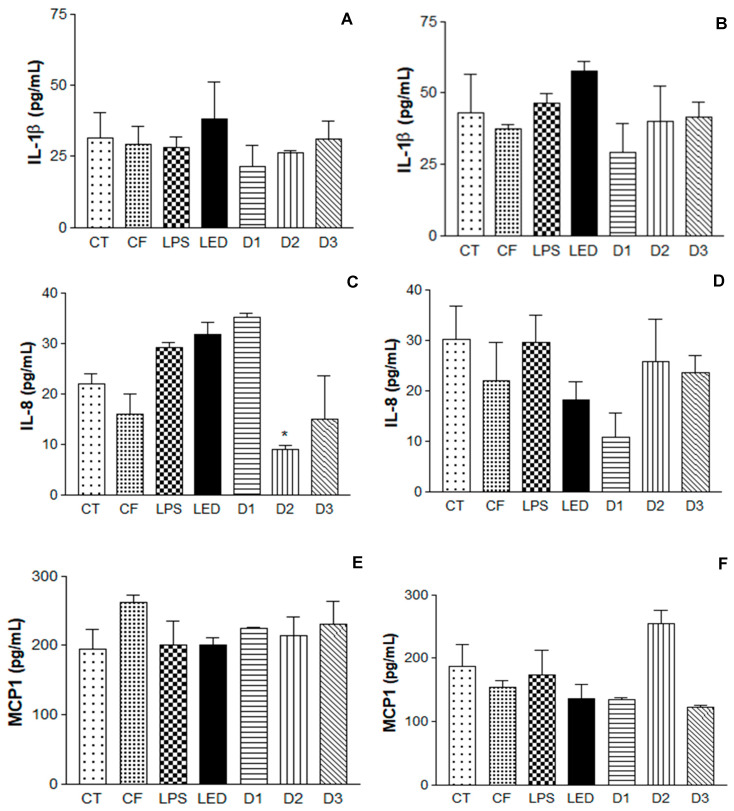
Enzyme-linked immunosorbent assay (ELISA) for the analysis of IL-1β, IL-8 cytokines, and macrophages MCP1 in human keratinocytes (HaCaT—cell line service) where (**A**,**C**,**E**) is the analysis 6 h after irradiation and (**B**,**D**,**F**) is the analysis 24 h after irradiation, where CT = control; LPS = lipopolysaccharide; LED = irradiation dose of 10 J. cm^−2^; D1 = QZ/NC irradiation dose of 1.0 J.cm^−2^; D2 = QZ/NC irradiation dose of 5 J.cm^−2^; D3 = QZ/NC irradiation dose of 10 J.cm^−2^; CF = formulation control (QZ/NC 15 μg.mL^−1^). Statistical significance was determined using the one-way ANOVA test, with a 95% significance level, followed by Dunnett’s post test (* *p* < 0.05).

The results obtained are summarized in Figure 6. It is possible to observe the induction of the inflammatory process, where the LPS levels were significant, leading to the expression of cytokines IL-1β, IL-8, and MCP1, as well the photodynamic effect of the QZ/NC and LED (460 nm) inducing the reduction in the cytokine level compared to the action of LED alone.

## 3. Discussion

Nanomaterials’ characteristics such as particle size, polydisperse index, and zeta potential are extremely important, directly influencing the biological environment and the effects of toxicity, cellular uptake, and permeability, which are fundamental for pharmaceutical applications [43]. Particle size especially is fundamental for the development of effective nanomaterials for topical application, considering the cutaneous permeation, where particles with submicron size tend to penetrate in a facilitated manner through the skin, reaching the systemic circulation [44,45]. Within the 61-day post-synthesis period, QZ/NC and Empty/QZ remained stable within the analyzed parameters, with a particle size between 100 and 150 nm for both formulations with no statistical significance (*p* value = 0.13), and a PdI between 0.4 and 0.5; however, 97.9% of the particles remained in the predominant average size, indicating a monodisperse system with variation relative to the larger particle distribution (Table 1). The zeta potential values remained within the proposed stability range within the analysis period (+30 mV, −30 mV). The surface characteristics, indicated by the zeta potential, influence the biological performance and safety of the nanomaterial, determining its biodistribution and special stability and, consequently, indicating the drug release in biological targets [46]. AFM analysis showed spherical particles, smaller than the values presented by the DLS technique (Figure 1), due to the difference in the technique, once DLS measures the particle’s hydrodynamic radius or solvation radius. The analysis confirms the average particle size expected for nanostructured polymeric systems and shows the characteristic spherical morphology, with excellent correlation with the hydrodynamic sizes obtained by the DLS technique, with no deformations on the surface, indicating a homogeneous particle formed only by the PLGA, which covers the nanocapsules [27].

The 3D fluorescence profile (Figure 2) showed no significant changes in the fluorescence profile of free QZ when nanoencapsulated, with no reaction of the active with the components used to prepare the nanostructured system, promoting support for the use of QZ/NC in future biological tests. Previous studies carried out with QZ in a spray-dryer nanosystem have shown favorable results and in agreement with the one presented, where in the nanoparticles with QZ, following encapsulation, the spectral profile remained unchanged, with a high association rate (72.3%), which is satisfactory for subsequent in vitro studies [47]. Considering the application in PDT, maintaining the photophysical properties of the PS in the ground state is extremely important, ensuring absorption in the visible region of the electromagnetic spectrum at the optimal excitation wavelength. 

Cytotoxicity studies are extremely important and necessary in the drug development and synthesis of new medicines, assessing the in vitro biological compatibility profile for biological application. There are also tests that help determine important nanotechnology development parameters, such as IC10 and IC50, which are exactly the inhibitory concentrations that induce a 10% and 50% reduction in cell viability, respectively. The cytotoxicity assay developed initially with NIH/3T3 cells (Table 2) showed biocompatibility at the lowest concentrations for all the samples tested (QZ/NC, Unloaded NC, and free QZ), without statistical significance and in accordance with the ISO 10993-5 (cytotoxicity effect will only be considered for cell viability below 75%). The concentration of 15 μg.mL^−1^ of QZ/NC was chosen for the development of the sequential investigative biological assays, considered the in vitro safety threshold. Further studies with HaCaT cells (Figure 5) showed cytotoxicity only at the higher concentrations of QZ/NC (50 µg.mL^−1^), showing compatibility in the specific concentration range (2.5–35 µg.mL^−1^), making it a potent candidate for use in protocols with biological application and future studies for the evaluation of photodynamic activity in models of inflammatory processes in vitro.

The processes of cell permeation and internalization are influenced by several variables, such as the size of the nanomaterial chosen, the concentration, pharmaceutical form, surface characteristics, and the internal structure [48]. The greatest limitations involved in the development of drugs for topical and transdermal application is the permeation barrier provided by the stratum corneum of the skin, composed of rigid layers rich in keratin and lipid intercellular matrix, which directly influences the therapeutic result [49,50]. The fluorescence analyses, in the internalization (Figure 3) and permeation (Figure 4) tests, showed higher internalization for QZ/NC compared to free QZ and permeation of QZ/NC in all the layers of the three-dimensional model. Fluorescence signals characteristic of the QZ/NC were observed, which is one of the main desired effects and justifies the use of nanotechnology for biological applications. Quantitative assessment of the fluorescent signals showed only statistical significance between the QZ/NC and free QZ, suggesting impaired permeability of free QZ through the skin model barrier. Previous studies analyzing the permeation of nanoparticles in human cadaver skin have observed an increase and improvement in the permeation of nanostructured compounds [49]. Also, considering the structure of the skin in inflammatory diseases such as psoriasis, where the hyperproliferation of keratinocytes and the inflammatory process make the skin rigid and impermeable, the use of polymeric nanoparticles is highly applicable, since they promote greater bioavailability and permeability in the stratum corneum of the skin [10].

To assess the QZ/NC efficiency, the inflammatory process in vitro using keratinocyte cells was developed (Figure 6). Upon triggering the inflammatory process in vitro, inflammatory mediators begin the production of cytokines, which are essential in driving the inflammatory response [51]. In the analysis of the cytokines IL-1β and IL-8, after 24 h of irradiation, the formulation had no influence on the level of cytokines, further corroborating the hypothesis that the association of the photosensitizer with the action of the LED is more effective (photodynamic effect). Regarding the energy doses used, the results varied depending on the cytokine analyzed in relation to the time observed. IL-1β, 6 h after irradiation, showed no statistically significant difference between the doses analyzed. However, D1 had a considerable reduction in cytokine expression after 24 h. IL-8, 6 h after, D2, and D3 showed significant differences when compared to the LPS control. After 24 h, D1 had the most significant difference in the group. MCP1 at 6h after irradiation showed no difference between doses. However, after 24 h, D1 and D3 showed a reduction in the expression of this cytokine. 

PDT showed greater potential, compared to LED therapy (LED use only) and the QZ/NC formulation applied without any irradiation. Previous studies have analyzed the action of PDT as a therapeutic method through the production of cytokines, observing an increase in pro-inflammatory cytokines (IL-1β, IL-8, IL-6, and MCP1), highlighting that the action of PDT has three phases: generation of ROS, expression of local endothelial damage, and the mechanisms of the disease, so patients who responded to PDT had their levels of pro-inflammatory cytokines increased, as a response to the inflammatory process [52,53,54]. Also, in comparison with PDT and LED therapy, PDT proved to be more effective in reducing cytokine expression 24 h after irradiation, when compared to the action of LED alone (therapeutic LED effect) [55,56]. The photodynamic effect of QZ has been reported previously, presenting the efficiency of a singlet oxygen formation of 0.23 and quantum yields of intersystem of 0.3, showing the generation of singlet oxygen is based on the Type II reaction, which favors its use in PDT, since this reaction leads to the production of reactive singlet oxygen [57,58,59]. Previous studies on anthraquinones and QZ have demonstrated the photodynamic effects of these compounds, generating singlet oxygen and leading to cell death, confirming the efficacy of photodynamic therapy in in vitro protocols [27,47,60]. Considering the cytokines analyzed above are directly linked to the inflammatory process of autoimmune and inflammatory diseases, these results show PDT as an effective treatment with potential to be explored in the future.

## 4. Materials and Methods

### 4.1. Development of Polymeric Nanocapsules Loaded with Quinizarin

Polymeric NCs (QZ/NC) were prepared using the nanoprecipitation method as described by Siqueira-Moura et al. [61], at the concentration of 0.1 mg.mL^−1^ QZ. The coating co-polymer poly (L-lactic acid-co-glycolic acid)—PLGA 50:50 (0.75% *w*/*v*) and lecithin (high-purity soy phosphatidylcholine) at 1.75% *m*/*v* were dissolved in 15 mL of acetone at 40 °C (characterizing the organic phase) and slowly added by dripping at the rate of 1.0 g.s^−1^, under the aqueous solution, containing Pluronic F-68 (1.25% *m*/*v*) in 30 mL of water under moderate and constant stirring (250 rpm) at 40 °C. The emulsification and interfacial coating process was started after phase mixing directly in a 250 mL jacketed reactor coupled to a thermostatic bath under constant circulation. Subsequently, the organic solvent was removed to finalize the nanoemulsification process, through the route-evaporation step at 40 °C and 225 rpm rotation under reduced pressure, obtaining a final formulation volume of 10 mL. Storage was carried out at 7 °C. 

### 4.2. Characterization of Particle Size, Polydispersity Index (PdI), and Zeta Potential

The physicochemical analyses were performed following the standard operating procedures of the equipment Zetasizer^TM^, model Nano ZS90 Malvern, (Malvern Panalytical Ltd, Malvern, UK) obtaining parameters for the particle size, polydispersity index (PdI), and zeta potential.

### 4.3. Atomic Force Microscopy

Atomic force microscopy allows the topographical surface of nanomaterials to be analyzed. Images of the QZ/NC were taken at room temperature using a Shimadzu Scanning Probe Microscope model SPM-9600 (San Jose, CA, USA). The sample was prepared on a mica surface, where a drop (5.0 µL) of the formulation was deposited. The images were obtained using the intermittent contact method, with a 124 µm long cantilever, operating at a resonance frequency in the range of 324–369 KHz, rigidity of 34–51 N/m, and constant force.

### 4.4. Development of Three-Dimensional Fluorescence Emission Spectroscopy UV/Vis

The 3D fluorescence emission spectra of QZ in acetonitrile and after nanoencapsulation (QZ/NC) were obtained as part of the spectroscopic characterization. Analyses were carried out using a fluorimeter (Shimadzu model RF-6000, San Jose, CA, USA) with the following parameters: emission wavelength (525–685 nm); excitation wavelength (400–520 nm); data interval: 2nm; excitation band: 1.5 nm; and emission band: 3 nm.

### 4.5. Cell Culture

The dermal fibroblast (HDFs—ATCCTM PCS-201-010™), adipose-derived stem cells (ADSCs R7788115—Thermo Fisher Scientific, Altrincham, Cheshire, UK), human epidermal keratinocytes (NHEKs—Thermo Fisher Scientific C0015C), immortalized human keratinocyte (HaCaT—cell line service), and commercial murine fibroblast (NIH/3T3—ATCCTM CRL-1658™) cell lines were cultivated using the standard cultivation protocol. The cell lines were cultured in an incubator with 5% CO_2_ at 37 °C, and the culture was conducted until the confluence stage was reached, where the cells were used for the subsequent assays.

### 4.6. Cellular Uptake

Immortalized human keratinocyte lineage cells (HaCaT—cell line service) were plated in 24-well plates (3 × 10^4^ cells.well^−1^) and maintained at 37 °C in an atmosphere containing 5% CO_2_ for 24 h. Subsequently, QZ/NC and free QZ were placed at a concentration of 15 µg.mL^−1^ at three different times (3 h, 6 h, and 24) in order to analyze the best time for cell internalization. After the mentioned times, the respective plates were washed with PBS and quantified using the EnSpireTM microplate reader (PerkinElmer, Shelton, CT, USA) where the fluorescence intensity was analyzed.

### 4.7. Cytotoxicity Assay Using Resazurin Test

NIH/3T3 and HaCaT cells were seeded into a 96-well plate (5 × 10^3^ cells.well^−1^); after 24 h, free QZ and QZ/NC in range concentration of 2.5−70 μg.mL^−1^ using mean DMEM for dilutions was added and incubated for 3 h for NIH/3T3 and 24 h for HaCaT as suggested by ISO 10993-5. After 24 h of treatment, 20 μL of Resazurin solution (25 μg.mL^−1^ in PBS) and 180 mL of DMEM were added and incubated for 4 h. Afterwards, the wells’ analyses were carried out in the microplate reader EnSpireTM (PerkinElmer, USA) in 570 nm and the basal absorbance was corrected in 590 nm. The percentage of viable cells was calculated following Equation (1).

Viable cells (%) = (O.D.sample/O.D.control) × 100(1)

### 4.8. Permeation Study in a New 3D Bioprinted Skin Equivalent: Suspended Layer Additive Manufacturing (SLAM) Method

The 3D skin equivalents and the printing media were prepared as described by Moakes et al. [33] following the method of suspended layer additive manufacturing. The layers were prepared from a blend of collagen to pectin: papillary layer (2:1 blend containing 3 × 10^6^ cells.mL^−1^ (HDFs—passage 8); reticular layer (2:1 blend containing 1.5 × 10^6^ cells.mL^−1^ (HDFs—passage 7); and 1:1 blend containing 5 × 10^5^ cells.mL^−1^ (ADCSs—passage 3). The construct’s G-code was sent to the printing program to print a tri-layer with the following dimensions with a cylindrical shape: A = 225 mm^2^; H = 1.2 mm in a 0.5% *w*/*v* Agarose fluid gel support bath. Subsequently, the constructs were gelled through the addition of a 200 mmol.L^−1^ CaCl_2(aq)_ solution to the support bath, followed by 30 min incubation at 37 °C. The constructs were then transferred into clean wells for culture at 37 °C and 5% CO_2_ with supplemented DMEM medium (10% *v*/*v* Fetal Bovine Serum, 1% *v*/*v* PenStrep; Sigma-Aldrich, Gillingham, UK) for 14 days. Keratinocyte cells (NHEKs 2.5 × 10^5^ cells.mL^−1^—passage 4) were placed on top of the constructs and cultured for another 7 days, forming the epidermal layer. The QZ/NC, at a concentration of 15 µg.mL^−1^, defined as the safety limit, and free QZ (15 µg.mL^−1^), both suspended in DMEM, were added to the top of the equivalents (80 µL), inside a cylindrical support, which ensured that the sample would be concentrated at the top of the construct; a control was also prepared with DMEM only. After 24 h, cross-sections were made, and the samples were analyzed using a SPARK microplate reader (TECAN, Männedorf, Switzerland) and a Cytation imaging reader (BioTek, Agilent, Santa Clara, CA, USA) where the fluorescence intensity was observed.

### 4.9. Induction of Inflammatory Process in HaCaT Cells and Application of Photodynamic Therapy

HaCaT cells were used as a biological model for the development of the inflammatory model. The cells were cultured as described in 2.7 and plated in 24-well plates. The cells were induced with bacterial lipopolysaccharide (LPS) at a concentration of 10 µg.mL^−1^ and incubated for 24 h—37 °C, 5% CO_2_. After 24 h, QZ/NC was added at the concentration of 15 µg.mL^−1^ and incubated for 24 h—37 °C, 5% CO_2_. Subsequently, the cells still with LPS and QZ/NC were submitted to PDT, where a 24-well LED tabletop photoactivation system (IrradLED/Biopdi) emitting at 470 nm with 37.2 mW optical power was used. The energy densities (fluences) used were as follows: 1.0 J.cm^−2^ (D1), 5.0 J.cm^−2^ (D2), and 10 J.cm^−2^ (D3) for the respective exposure times of 27 s, 134 s, and 269 s. Two plates were made for post-irradiation analysis.

### 4.10. Enzyme-Linked Immunosorbent Assay (ELISA)

For in vitro assays, IL-1β, IL-8, and MCP-1 levels of cell supernatants were detected using the commercially available human quantikine ELISA kit (BD Biosciences, San Jose, CA, USA), and according to the manufacturer’s instructions, following the protocol previously described by Cardoso et al. [62], being a sequential experiment of the one presented in 2.9. Briefly, 100 μL of each of the standards, controls, and samples was loaded into 96-well polystyrene microplate wells, containing 100 μL of assay diluent in triplicate. After incubation for 2 h, the microplates were washed 4 times. Then, 200 μL of specific conjugate solutions was added to each well for 2 h, washed, and 200 μL of a substrate solution was added. After 20 min of incubation, 50 μL of the stop solution was added and the optical density was read at 450 nm using a microplate reader (Molecular Devices, Sunnyvale, CA, USA) with a correction wavelength of 540 nm.

### 4.11. Statistical Analysis

Statistical analysis was performed using one-way analysis of variance (one-way ANOVA), with Tukey’s test (cytotoxicity) and Dunnett’s test (ELISA) as post-test for multiple comparisons analysis between the groups (* *p* < 0.05; ** *p* < 0.001). All statistical analyses were carried out with *n* = 3.

## 5. Conclusions

A novel therapeutic approach combining nanomedicine and photodynamic therapy (PDT) was successfully developed for addressing inflammatory skin diseases. The nanomaterials created exhibited submicron size and maintained stability throughout the analysis period, demonstrating promising photophysical properties attributed to the presence of quinizarin. Spectroscopic studies confirmed that the photophysical characteristics of the photosensitizer remained unchanged whether in its free form or nanoencapsulated. In vitro biological assays revealed low toxicity levels, meeting the essential safety criteria for nanomaterial applications. Permeation studies using a 3D skin model, along with cellular uptake analyses, demonstrated that QZ/NC effectively permeates skin-like models and is incorporated intracellularly, thereby enhancing drug delivery efficiency. The in vitro model of the inflammatory process was successfully established, followed by PDT application and subsequent immuno-enzymatic analysis. Notably, PDT exhibited significant therapeutic effects compared to LED treatment and QZ/NC alone, yielding highly favorable outcomes. These findings suggest that the developed model, combined with synthesized nanocapsules and PDT, represents a promising new therapeutic strategy for anti-inflammatory therapy.

## Figures and Tables

**Table 1:** Data of average * particle size, PdI, and zeta potential of QZ/NC and Unloaded/NC.

Nanomaterial	Average Particle Size (nm)	PdI	Zeta Potential (mV)
QZ/NC	103.9 ± 34.5	0.4 ± 0.03	−31.8 ± 0.723
Unloaded/NC	137.2 ± 46.1	0.5 ± 0.06	−35.1 ± 1.107

* *n* = 3.

**Table 2:** Cellular viability * obtained from in vitro cytotoxicity assay of QZ/NC, Unloaded/NC, and free QZ on murine fibroblast cell lines NIH/3T3 (ATCC^®^ CRL-1658) at concentrations of 2.5; 5; 15; 25; 50; and 70 μg.mL^−1^ (QZ/NC), 50% (*v*/*v*) Unloaded/NC and free QZ at concentrations of 50 and 70 μg.mL^−1^. Statistical significance was determined using the one-way ANOVA test at 95% significance level, followed by Tukey post-test for multiple comparisons (* *p* < 0.05, ** *p* < 0.001).

Sample/Concentration (µg.mL^−1^)	3T3—Cellular Viability (%)	Significance Level (* *p* < 0.05, ** *p* < 0.001)
Control	100	-
Unloaded Nanocapsule	81.43	-
Free QZ 50	100.06	-
Free QZ 70	91.30	-
QZ/NC 2.5	98.32	-
QZ/NC 5.0	100.92	-
QZ/NC 15.0	96.69	-
QZ/NC 25.0	69.82	*
QZ/NC 50	9.66	**
QZ/NC 70	9.02	**

* *n* = 3.

## Data Availability

The original contributions presented in the study are included in the article, further inquiries can be directed to the corresponding author.
